# Surgical treatment of posterior cruciate ligament lesions does not cause growth disturbances in pediatric patients

**DOI:** 10.1007/s00167-018-5308-5

**Published:** 2018-11-21

**Authors:** Helmut Wegmann, Sophie Janout, Michael Novak, Tanja Kraus, Christoph Castellani, Georg Singer, Holger Till

**Affiliations:** 1 Department of Pediatric and Adolescent Surgery, Medical University of Graz, Auenbruggerplatz 34, 8036 Graz, Austria; 2 Department of Pediatric Orthopedics, Medical University of Graz, Graz, Austria

**Keywords:** Pediatric, Adolescent, Surgical management, Posterior cruciate ligament, Outcome, Physis

## Abstract

**Purpose:**

The aim of the present study was to describe epidemiology, management and outcome of pediatric and adolescent patients with posterior cruciate ligament (PCL) injuries.

**Methods:**

Sixteen patients of less than 18 years of age with 7 PCL avulsion fractures and 9 PCL tears were included over a 10-year period. Trauma mechanism, additional injuries and treatment methods were analyzed. Follow-up examination included range of motion and ability to perform squats. Pedi-IKDC and Lysholm score were obtained and posterior shift was measured in kneeling view radiographs and compared to the contralateral side. Patients were grouped into pediatric patients with open physes at the time surgery and adolescent patients with closing or closed physes. In case of open physes, growth disturbances were assessed.

**Results:**

Six of the treated patients (median age 12.5 years, range 10–13) had open physes at time of surgery. Five of those sustained avulsion fractures and treatment consisted of open reduction and screw fixation in four cases and graft reconstruction in one case. One patient sustained a PCL tear and underwent graft reconstruction. Follow-up at a median of 71.5 months (range 62–100) did not reveal any growth disturbances. Median Pedi-IKDC was 71.9 (range 51.7–92.1), median Lysholm score was 81.5 (range 66–88) and median posterior shift difference was 2.5 mm (range 0–11). The remaining 10 patients (median age 16 years, range 14–17) had closing/closed physis at the time of operation. Two patients presented with avulsion fractures treated with open reduction and screw fixation and 8 patients sustained PCL tears treated with graft reconstruction. At a median follow-up of 69.5 months (range 11–112), median Pedi-IKDC was 86.8 (range 36.8–97.7), median Lysholm score was 84.0 (range 45–95) and median posterior shift difference was 4 mm (range 0–15).

**Conclusions:**

In our small number of pediatric patients with PCL injuries, open reduction and epiphyseal screw fixation of displaced avulsed fractures and steep tunnel drilling in case of PCL reconstruction did not cause growth disturbances. Nevertheless, long-term functional impairment should be expected and close follow-up has to be recommended.

**Level of evidence:**

Therapeutic, Level IV.

## Introduction

Posttraumatic lesions of the posterior cruciate ligament (PCL) are rarer than those of the anterior cruciate ligament (ACL) [17]. The incidence of PCL lesions has been reported with a great variability ranging from 1 to 44% of all knee injuries [21]. Typically, patients with PCL lesions are older than those with ACL injuries [16]. Consequently, PCL lesions are uncommonly diagnosed in pediatric and adolescent patients [22].

Typical mechanisms causing PCL lesions include hyper-extension or hyper-flexion of the knee and posterior displacement of the tibia in relation to the femur while the knee is flexed (“dashboard injury”) [14]. In the majority of cases, PCL lesions are caused by a high-energy trauma resulting in concomitant damages to other structures of the knee (in about two-thirds of PCL cases) and to other body regions (in 92.5% of PCL cases) [16].

Although conservative treatment of an isolated PCL lesion is possible, it is usually associated with residual laxity and subsequently a higher incidence of osteoarthritis in the medial femoro-tibial compartment [19, 21].

Overall, reports on epidemiology, treatment and outcome of PCL lesions of pediatric and adolescents patients are confined to case reports or small case series [12, 20, 22]. Kocher and coworkers have reviewed 15 operatively treated pediatric and adolescent patients with a small subgroup of 7 skeletally immature patients. However, in 5 of these 7 patients, the physes were judged as impotent for further growth and the patients underwent allograft PCL reconstruction. The two remaining patients with open physes were treated with femoral suture anchors [12]. Another series has reported 6 patients with open physes treated with extraphyseal PCL reconstruction [22]. Shah et al. have published three pediatric cases of extraphyseal PCL reconstruction with maternal allograft and reported excellent results [20].

The aim of the present study was to describe epidemiology and management of a series of pediatric and adolescent patients with PCL lesions. The outcome of a group of adolescent patients following surgical management of PCL lesions is demonstrated. Additionally, a subgroup of pediatric patients with open physes following transphyseal reconstruction and epiphyseal screw fixation is described.

## Materials and methods

In this single-center retrospective cohort study with prospective follow-up, all patients of less than 18 years of age (at the time of the accident) with PCL lesions were included covering a 10-year period from 1.1.2005 to 31.12.2014. Information concerning gender, age and height at time of surgery, trauma mechanism, the nature of the PCL lesion (avulsion fracture or tear), concomitant injuries and treatment method was collected retrospectively from medical files and radiological images.

All patients were invited for a clinical and radiological follow-up examination. The range of motion of the affected knee joint was measured with a goniometer and the ability to perform deep squats was assessed. The Pediatric International Knee Documentation Committee (Pedi-IKDC) Score and Lysholm Score were evaluated [5, 23]. In skeletally immature patients with open physes on preoperative radiographs, body height was obtained at time of follow-up to document growth after operation.

At follow-up, the radiological examination consisted of bilateral conventional X-rays (non-weight-bearing anterior–posterior and lateral views). If present, osteoarthritis was classified according to Kellgren–Lawrence [11]. Tibial and femoral angles (anatomical lateral distal femoral angle—aLDFA; anatomical posterior distal femoral angle—aPDFA; medial proximal tibial angle—MPTA; anatomical posterior proximal tibial angle—aPPTA) were measured and compared to the contralateral side to exclude postoperative growth disturbance in case of open physes at time of surgery. Additionally, leg length discrepancy was assessed clinically. A bilateral kneeling view [7] was obtained to determine posterior shift using the method described by Jacobsen [8].

Patients were grouped into pediatric patients with open physes at the time surgery and adolescent patients with closing or closed physes.

The study was approved by the institutional ethics committee of the Medical University of Graz (27-314 ex 14/15). Prior to the examination informed written consent was obtained from the patients and/or legal guardians.

### Surgical technique

**Fig. 1:**
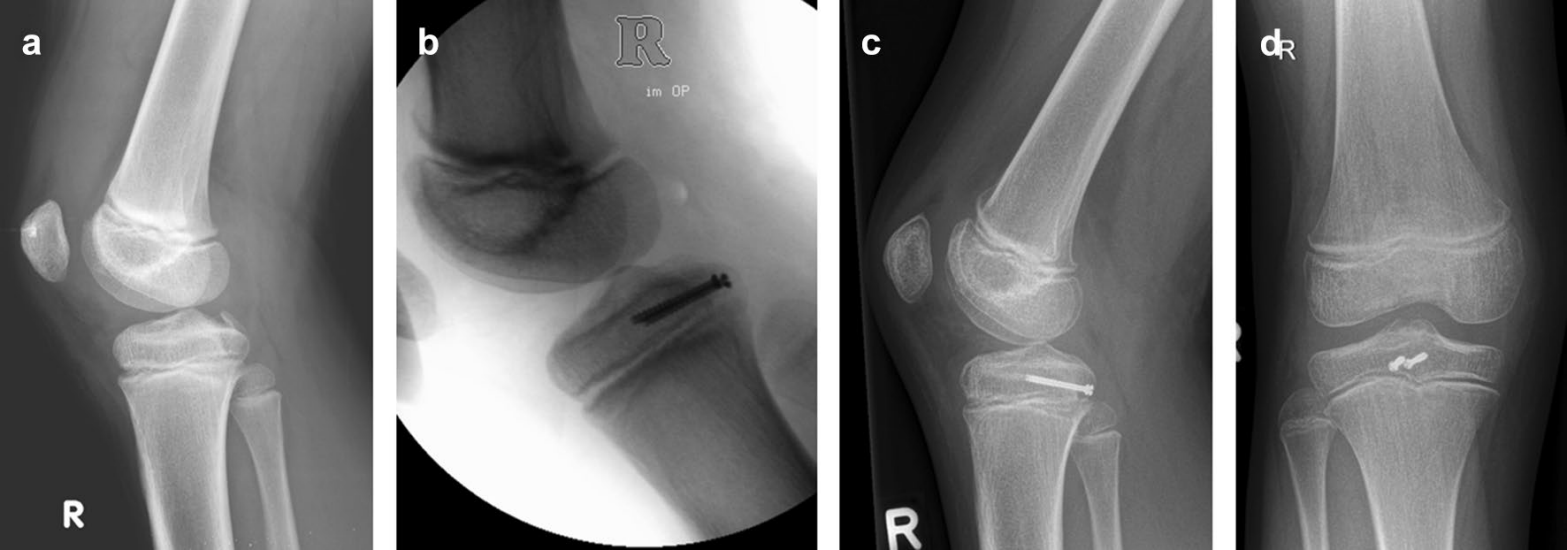
Example of a 10-year old patient with a displaced avulsion fracture of the PCL sustained in a road traffic accident (**a**). The injury was treated with open reduction and screw fixation (**b**). Postoperative radiographs showing anatomic reduction of the fracture (**c, d**)

Open reduction and screw fixation was performed in cases of displaced avulsion fractures and was achieved with retention stiches, controlled radiologically and maintained with one or two K-wires, depending on the size of the displaced fragment. 3 mm cannulated screws were inserted. In case of open physis, extraphyseal positioning was verified fluoroscopically. An example of a 10-year old patient with a displaced PCL avulsion fracture is shown in Fig. 1. Generally, patients after screw fixation were placed in a non-weight-bearing long leg cast for 6 weeks followed by physical therapy with gradually increasing weight-bearing aiming for full weight-bearing 8 weeks postoperatively.

In cases of arthroscopic graft reconstruction, two different techniques for tunnel drilling were used: (1) femoral and tibial inside-out retrograde drilling using Arthrex Retro-Flip cutters (Arthrex, Naples, FL, USA) or (2) antegrade femoral and tibial tunnel drilling with cannulated drill bits. Reconstruction was performed with gracilis/semitendinosus, quadriceps graft or bone patellar tendon bone (BPTB) according to the pattern of the injury. In patients with open physes steep tunnel drilling with a minimal aiming angle of 60° was performed to minimize injury to the physis by creating a small cross-sectional area of the tunnel at the level of the physis. Graft fixation was achieved either with absorbable interference screws or with tight rope fixation (Arthrex, Naples, FL, USA). A postoperative long leg plaster was applied for 2 weeks followed by a PCL brace for additional 4 weeks. Non-weight-bearing was advised for 6 weeks and thereafter physical therapy for knee mobilization was initiated. Full weight-bearing was allowed 8 weeks postoperatively.

## Results

From 2005 to 2014, 16 pediatric and adolescent patients (*n* = 8 male, *n* = 8 female; median age 14 years (range 10–17 years)) with lesions of the PCL were surgically treated at the Department of Pediatric and Adolescent Surgery of the Medical University of Graz. The majority of lesions was caused by road traffic accidents (*n* = 11; 69%). The lesions consisted of 7 PCL avulsion fractures with a median displacement of 10 mm (range 7–21 mm) and 9 PCL tears.

For further analysis, patients were divided into pediatric patients with open physes and adolescent patients with closing or closed physes. Table 1 displays etiology, PCL lesion (avulsion fracture or tear) and concomitant injuries of the patients.

**Table 1:** Etiology, diagnosis, concomitant injuries of 16 patients with PCL injuries. Patients are grouped according to the status of the physis

	Open physes (*n* = 6)	Closed physes (*n* = 10)
Etiology	*n* = 4 RTA *n* = 1 fall *n* = 1 SA	*n* = 7 RTA *n* = 2 SA *n* = 1 fall
Lesion	*n* = 5 AF *n* = 1 PCL tear	*n* = 8 PCL tear *n* = 2 AF
Additional knee injury^a^	*n* = 2 MCL rupture *n* = 1 LCL rupture *n* = 1 ACL avulsion	*n* = 6 ACL lesion *n* = 3 meniscal tear *n* = 3 PLC lesion *n* = 2 MCL rupture *n* = 1 LCL rupture
Additional extraarticular injuries^a^	n = 1 calcaneus^b^ n = 1 tibial plateau^b^	*n* = 3 peroneal nerve lesion *n* = 2 compartment syndrome *n* = 2 popliteal artery dissection *n* = 1 tibial plateau^b^

*RTA* road traffic accident, *SA* sports accident, *AF* avulsion fracture, *ACL* anterior cruciate ligament, *PCL* posterior cruciate ligament, *LCL* lateral collateral ligament, *MCL* medial collateral ligament, *PLC* postero-lateral corner

^a^Some patients sustained multiple lesions

^b^Fracture

### Patients with open physes

Six patients (median age 12.5 years, range 10–13 years, *n* = 5 males) were skeletally immature with open physes at time of surgery. While four of these patients sustained additional knee injuries, two children presented with additional extraarticular injuries (Table 1).

Five patients were diagnosed with displaced avulsion fractures of the PCL and one patient sustained a PCL tear. Of the five patients with avulsion fracture, four patients were treated with open reduction and extraphyseal screw fixation, and one with transphyseal quadriceps tendon graft. In this patient the avulsion fragment was too small for screw fixation. The remaining patient with PCL tear was treated with transphyseal gracilis and semitendinosus graft.

All physes were closed at follow-up examination (median 71.5 months, range 62–100). While the median body height at the time of surgery was 144 cm (range 137 to 157 cm), median patients' body height at follow-up was 172.5 cm range (167–182 cm). Thus, median growth after surgery was 27 cm (range 20–34 cm).

Clinical values and scores at follow-up are shown in Table 2. Plain radiographs showed signs for osteoarthritis grade 1 in one patient. Growth disturbances as measured by aLDFA, aPDFA, MPTA and aPPTA were not found. Additionally, none of the patients developed leg length discrepancies.

**Table 2:** Clinical findings and scores at follow-up of 6 patients with open physes and 10 patients with closed physes

	Open physes [median (range)] (*n* = 6)	Closed physes [median (range)] (*n* = 10)
Extension injured knee [°]	5 (0–5)	2.5 (0–15)
Flexion injured knee [°]	135 (110–140)	125 (80–140)
Deep squats (> 90°) possible	3/6	3/10
Pedi-IKDC	71.9 (51.7–92.1)	86.8 (36.8–97.7)
Lysholm score	81.5 (66–88)	84 (45–95)
Posterior shift^a^ [mm]	2.5 (0–11)	4 (0–15)

^a^Posterior shift is shown as the difference to the uninjured knee as measured in kneeling view

### Patients with closing or closed physes

10 patients (median age 16 years, range 14–17 years, *n* = 3 males) were prepubescent with closing physes or skeletally mature at time of surgery. Eight of these patients presented with additional injuries to the knee and half of the patients sustained additional extraarticular injuries (Table 1).

Two patients were diagnosed with displaced avulsion fractures of the PCL. Both were treated with open reduction and screw fixation; one required revision surgery for persisting instability and was treated with a bone patellar tendon bone (BPTB)-graft. The remaining eight patients sustained PCL tears and were treated with graft reconstruction. Four patients were treated with a gracilis/semitendinosus tendon graft. One of these patients suffered from a re-rupture and required BPTB-graft in the later course. Three patients were primarily treated with quadriceps tendon graft and one patient was treated with a BPTB-graft.

Patients were reexamined after a median follow-up of 69.5 months (range 11–112 months). Clinical outcome and scores at follow-up are depicted in Table 2. Plain radiographs showed signs for osteoarthritis grade 1 in 5 patients and grade 2 in 2 patients.

## Discussion

The most important finding of the present case series was that in 6 patients with open physes no growth disturbance was seen at follow-up examination after surgical treatment of PCL injury. Additionally, posterior shift was increased in both children with open and adolescents with closed physes. The majority of PCL injuries derived from high-energy trauma and led to multiple injuries of both the knee as well as extraarticular structures. Avulsion fractures were predominantly encountered in patients with open physes and PCL tears in patients with closing or closed physes confirming previous reports [3].

Injuries of the PCL are rarely diagnosed in patients with open physes. Therefore, information concerning postoperative growth disturbances is confined to small case series. In the report of Kocher and coworkers two patients with open physes were treated with femoral suture anchors without subsequent growth disturbances [12]. Sorensen et al. and Shah et al. have treated their pediatric patients with femoral and tibial extraphyseal graft reconstruction [20, 22]. However, placing the tibial tunnel distal to the physis leads to distal migration of the bone tunnel during growth and as a consequence the reconstructed PCL might elongate over time [22]. In contrast, in the two patients with open physes treated with graft reconstruction of our series, the tibial tunnels were drilled across the physes aiming at the tibial footprint of the PCL. The risk of growth disturbance is determined by the extent of damage to the cross-sectional area of the physis [6] and depends on the remaining growth potential of the patient [18]. While drill holes of 6 mm or smaller did not cause growth disturbance [15], tunnels of 12 mm or more have been shown to lead to growth disturbances [13]. To minimize potential damage to the physis, the tibial tunnels in our series were drilled steeply in a 60° angle to avoid damage of a greater cross-sectional area of the physes. Moreover, the femur tunnel was drilled strictly epiphyseally. Growth disturbances were not caused by this approach. This is consistent with two previous case reports demonstrating unremarkable growth after transphyseal PCL reconstruction in two pediatric patients [1, 3].

In case of displaced avulsion fractures, anatomical reduction and screw fixation of the avulsed fragment should be considered [2]. However, there is no information which amount of displacement allows conservative treatment in pediatric patients. Minimally displaced avulsion fractures might be successfully treated with quadriceps muscle strengthening [10]. In the present series, all six cases of avulsion fractures were considerably displaced (median 10 mm, range 7–21 mm). Therefore, operative treatment was chosen. In the four pediatric patients with open physes, all screws were applied epiphyseally (compare Fig. 1). One patient with open physis and tibial avulsion fracture was not eligible for screw fixation due to the small size of the osteochondral fragment and therefore had to be treated with tendon graft. This underlines the importance of preoperative diagnostics for proper planning of surgical treatment of PCL injuries in pediatric patients.

Injuries of the PCL usually result from high-energy trauma. Therefore, associated injuries of the affected knee as well as the lower extremity have been shown in up to 80% of the cases [4]. This is in line with our findings with 13 out of 16 patients of the whole collective suffering from concomitant injuries to either the knee or other extraarticular structures. Due to the complexity of these injuries, it is hardly possible to deduct a clear treatment algorithm from our data. Nevertheless, gracilis/semitendinosus graft was the first choice for PCL reconstruction. However, in cases of concomitant medial ligament injuries or ACL tears the quadriceps tendon was used to further not weaken the medial compartment or preserve tendons for further ACL reconstruction. BPTB-graft was generally preferred for revision surgery.

The posterior shift at follow-up in our patients is comparable to previous reports. Sorensen et al. and Shah et al. have reported only minor postoperative posterior shift in pediatric patients with single PCL injuries [20, 22]. On the other hand, Kocher and colleagues have reported that two-thirds of pediatric and adolescent patients treated for PCL lesions—including multiple injuries—had dorsal instability between 3 and 5 mm and 13% between 6 and 10 mm [12]. In our series, patients with the highest amount of posterior shift at follow-up were multiply injured.

Reported outcome scores for operatively treated PCL injuries in pediatric and adolescent patients seem to depend on the complexity of the injury. While favorable scores have been shown for single PCL lesions [9, 20, 22], multiple injuries have been shown to be associated with lower outcome scores comparable to our findings [12].

Limitations of the present study include its retrospective character. Furthermore, the number of patients is small and therefore no statistical analysis or comparison between the different treatment methods was possible. However, pediatric PCL lesions are very rare and thus data derived from small cohorts still add information to the treatment of these severe injuries. To overcome these drawbacks a prospective ideally multi-centric approach coupled with longer follow-up would be necessary. Nevertheless, growth disturbances were prevented by physes respecting techniques consisting of extraphyseal screw fixation in the case of avulsed PCL lesions and steep tunnel drilling in case of transphyseal reconstruction in the present series of pediatric patients.

## Conclusion

PCL lesions in pediatric and adolescent patients are infrequent and in case of concomitant multiple knee injuries challenging to treat. In case of open physis, open reduction and internal screw fixation for avulsion fractures and PCL reconstruction seems to be possible without causing growth disturbance. Due to the heterogeneity of associated lesions treatment regimens have to be tailored individually according to the injury pattern.

## Abbreviations

ACL: Anterior cruciate ligament
AF: Avulsion fracture
aLDFA: Anatomical lateral distal femoral angle
aPDFA: Anatomical posterior distal femoral angle
aPPTA: Anatomical posterior proximal tibial angle
BPTB: Bone patellar tendon bone
G/ST: Gracilis/semitendinosus
LCL: Lateral collateral ligament
MCL: Medial collateral ligament
mm: Millimeter
MPTA: Medial proximal tibial angle
Pedi-IKDC: Pediatric International Knee Documentation Committee
PLC: Posterior lateral corner
Quad: Quadriceps tendon
RTA: Road traffic accident
SA: Sports accident
